# The Role of Lithium Carbonate and Lithium Citrate in Regulating Urinary Citrate Level and Preventing Nephrolithiasis

**Published:** 2009-09

**Authors:** Xiaobo Zhang, Piyush Aggarwal, Xiaoming Li, Crystale Oakman, Zhiping Wang, Ronald Rodriguez

**Affiliations:** 1 *Department of Urology, the First Hospital of LanZhou University, LanZhou, China*; 2 *Department of Pathology, the Second Hospital of LanZhou University, LanZhou, China*; 3 *Department of Urology, the Second Hospital of LanZhou University, LanZhou, China*; 4 *The Brady Urological Institute, Johns Hopkins Medical Institutions, Baltimore, USA*

**Keywords:** kidney calculi, lithium, nephrolithiasis, urolithiasis, urinary citrate levels

## Abstract

Background and purpose: Urinary Citrate is an inhibitor of Calcium oxalate stone formation. It is reabsorbed in the proximal kidney through sodium dicarboxylate co-transporters (NaDC-1, NaDC-3) present in the renal tubular epithelium. Lithium (Li) is a known potent inhibitor of these transporters. We investigated the effect of lithium carbonate (LiC) and lithium citrate (LiCit) in regulating urinary citrate levels and preventing nephrolithiasis (NL) in the rat model. Experimental approach: We took 220 Wistar rats and induced nephrolithiasis in 130 of them by administering high doses of 5% ammonium oxalate (AmOx) for seven days and labeled them as Group B. Rest were labeled as Group A. Each group was then divided into 3 subgroups. First sub-group acted as control while other two were treated with either lithium carbonate (LiC) or lithium citrate (LiCit) for 21 days. Ten rats from each of the six sub-groups were randomly selected for sacrifice on 3^rd^, 7^th^ and 14^th^ day and additional 10^th^ and 21^st^ day from Li treated groups. Blood and urine samples were collected and analyzed on these days. The kidneys of the sacrificed rats were dissected and studied under light microscopy for crystal deposition (left kidney) and histological changes (right kidney). Key results: Urinary citrate levels were significantly increased in response to either LiC (*p*<0.001) or LiCit (*p*<0.001). Increased urinary citrate levels resulted in the reduction of calcium oxalate (CaOx) crystal deposition, kidney tubular dilatation and infiltration of inflammatory cell in the tubulo-interstitium. Conclusions and implications: Use of lithium salts might be a potentially useful approach in the prevention of recurrent NL.

## INTRODUCTION

Citrate is a tricarboxylic (TCA) acid and an intermediate in the TCA (Krebs) cycle. It is normally excreted in urine and naturally prevents crystallization by complexing with calcium (1) and inhibiting crystal growth and aggregation (2, 3). Hypocitraturia is found as an isolated abnormality in up to 10% and secondary to other abnormality in 20% to 60% of nephrolithiasis (NL) patients (4–6). Oral administration and local chemolysis using citrate salts have been evaluated to treat NL (7). Neither approach has widespread acceptance due to high dosing frequency, bitter taste, and GI side effects while a need for nephrostomy tube for the latter.

Hypocitraturia results from reabsorption of citrate in the proximal convoluted tubules (PCT) in the kidney (8). The glomerulus freely filters citrate, so urinary levels depend primarily on its reabsorption through the PCT. Citrate reabsorption across the brush-borders of PCT occurs due to the activity of the sodium dicarboxylate co-transporters (NaDC-1 and NaDC-3) (9). Plasma citrate is transported by sodium-citrate co-transporter (NaCT) into liver, testes and brain cells (10). The efficiency of these transporters plays a vital role in the regulation of body citrate levels. The excessive re-absorption of citrate from urine may be the underlying mechanism of idiopathic hypocitraturia (11).

We have identified an alternative method of enhancing urinary citrate excretion by using lithium. Li impedes the normal physiologic reabsorption of citrate in the proximal renal tubular epithelium by inhibiting NaDC-1 and NaDC-3 (12). It also facilitates NaCT, resulting in enhanced transport into the liver, brain and testes (10). Hence, Li treatment may have pleomorphic effects on serum and urinary levels of citrate. We show the efficacy of LiC or LiCit in the regulation of rat urinary citrate levels and demonstrated that such treatments result in a significant decrease in the development of NL (13).

## MATERIALS AND METHODS

### Animals

We used 220 male Wistar rats as approved by the local animal ethics committee. Each subject was housed in metabolic cages for collection of urine and controlled feeding. These rats were initially divided into 2 groups, A and B. Group A rats were fed with normal forage and tap water for 7 days to allow them to acclimate to the metabolic cages, while Group B rats were used as our model for NL and were instead fed with 5% AmOx mixed forage for a week so that they develop CaOx crystals in the kidney. Both of these groups were then subdivided into 3 groups each. One subgroup acted as control while other two were given either 36.4 mg LiC (Sigma-Alorich, USA; Li_2_CO_3_) or 94 mg LiCit (Sigma-Alorich, USA; Li_3_C_6_H_5_O_7_·4H_2_O) per day in two divided doses along with normal forage. All sub-groups had 30 rats each and were fed for 14 days except for Li treated sub-groups in NL model rats (group B2 and B3) which had 50 rats each and were fed for 21 days. (Table 1) All rats had free access to tap water. The LiC and LiCit were gavaged twice daily using 18 gauge angio-cath sheath directly into the stomach. The experimental dose of LiC and LiCit used for rats was determined from human clinical dose through pharmacological method.

**Table 1:** Schema of the experiment

	Acclimatisation (7 days)	Sub-Groups	N	Treatment (days)				
				3	7	10	14	21

Group A	Forage	Group A1 (Control)	30	Forage				
		Group A2 (LiC)	30	Forage + 1 ml of 18.2 mg/ml LiC				
		Group A3 (LiCit)	30	Forage + 1 ml of 47 mg/ml LiCit				
Group B	5% AmOx Forage	Group B1 (NL)	30	5% AmOx Forage				
		Group B2 (NL + LiC)	50	Forage + 1 ml of 18.2 mg/ml LiC				
		Group B3 (NL + LiCit)	50	Forage + 1 ml of 47 mg/ml LiCit				
	Total		220					

Rats were divided into two groups A & B. B group rats served as model for nephrolithiasis and were fed with 5% AmOx mixed forage. Samples were collected on day 3, 10 and 14 for groups 1 to 4 while additionally on day 7 and 21 for group 5 and 6. AmOx, Amonium Oxalate; NL, Nephrolithiasis; LiC, Lithium carbonate; LiCit, Lithium Citrate.

### Sample collection and processing

Ten randomly selected rats from each group were sacrificed on day 3, 7, and 14 and additionally on day 10 and 21 from group B2 and B3. Blood and 24 hour urine samples were collected from these rats. Urinary samples were processed after adding 0.1 g Thymol and 0.5 ml 12 N HCl in 100 ml to prevent bacterial digestion of citrate (14). Half of the samples (urine and blood) were used for citrate and oxalic acid assays while other half was used for biochemical parameters such as calcium, uric acid and pH. Both kidneys from sacrificed rats were dissected. The right kidney was fixed in 10% formalin for histological examination whereas the left kidney was used for crystal deposition examination by light microscopy.

Citrate levels in both plasma and urine were assayed with enzymatic methods. A 500 μl sample was filtrated through 0.22-μm cellulose filters (Millipore, Bedford, MA) before analysis and then 200 μl of it was mixed with 2 ml buffer solution (50 ml 100 mM Tris, 50 ml 0.2 mM ZnSO_4_, 7 mg NADH and 70 μl MDH (6MU/L)) followed by addition of 20 μl citrate lyase 14kU/L (about 1.6 g/L). The sample was measured for absorbance with a Spectronic 3000 Array (Milton Roy, NY) UV-vis spectrophotometer at 340 nm (15).

Plasma Lithium and other biochemical markers were measured by using Auto Biochemical Analysis Apparatus (SYNCHRON CX7 Beckman Coulter). The pH values were measured by a pH meter (Mettler Toledo 320-S, sensitivity 0.01). To analyze crystalluria, freshly collected urine samples were transferred to a Malassez cell and examined under a polarizing microscope (CX31-P, Olympus).

### Crystal deposition evaluation

Dissected left kidneys were evaluated under light microscopy for crystal deposition and was graded as follows: 0 = no crystal deposits; 1 = a small quantity of crystal deposits sporadically in the renal medullae; 2 = a moderate quantity of crystal deposits in the renal medullae and the papilla; 3 = a large quantity of calculi plaque in the renal medullae, the papilla and the cortex; 4 = a large quantity of calculi plaques complicated with hydronephrosis or renal abscess formation.

### Pathological examination

The renal cortex was stained with periodic acid-Schiff. 2μm paraffin sections were examined using light microscopy. The pathological alterations were graded as 0 = no visible lesions, 1 = mild dilation of tubules, tubulo-interstitial inflammatory infiltration, lesion area <20%; 2 = dilation of tubules, tubulo-interstitial inflammatory infiltration, lesion area <40%; 3 = severe dilation tubules, massive tubulo-interstitial inflammatory infiltration, lesion area >40%.

### Statistical analysis

The data was analyzed using the SPSS 13.0 software. The variables were compared using one-way analysis of variance (ANOVA) and turkey post-tests. Urinay and plasma citrate levels in Group B were analysed using two-way ANOVA with Bonferroni post-tests. A value *p*<0.05 was considered statistically significant.

## RESULTS

### Evaluation of citrate levels

**Figure 1:**
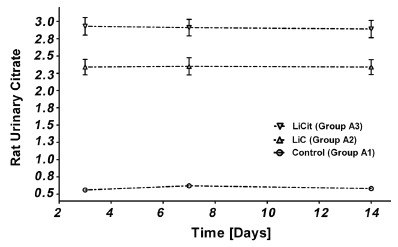
Urinary citrate Levels after administration of LiC and LiCit in group A rats. Urinary Citrate levels are increased with administration of LiC and LiCit as compared to untreated controls.

**Figure 2:**
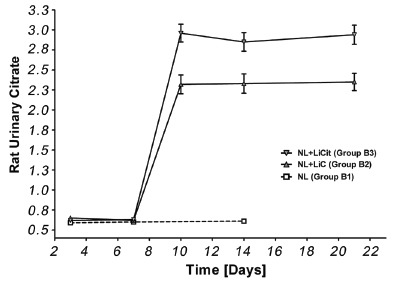
Urinary Citrate Levels after administration of LiC and LiCit in group B rats. Urinary Citrate levels increased significantly after 3 days of administration of LiC and LiCit compared to untreated NL model rats.

After three days of gastric gavage, urinary citrate levels were found to be fourfold [2.4 mmol/L (*p*<0.001)] and fivefold [3.0 mmol/L (*p*<0.001)] higher in group A rats treated with LiC (Group A2) and LiCit (Group A3) respectively when compared to control (Group A1). No further increase was observed with treatment time (Figure 1). In the rat model of AmOx induced NL (Group B), there was one week delay in the lithium-induced enhancement of urinary excretion of citrate (Figure 2), though otherwise the magnitude of increase in citraturia was same as in group a rats (*p*<0.001). Further, the greater increase in urinary citrate levels in LiCit treated sub-groups was significant when compared to LiC sub-groups. (P<0.001, Group B2 vs. B3; Group A2 vs. A3).

**Figure 3:**
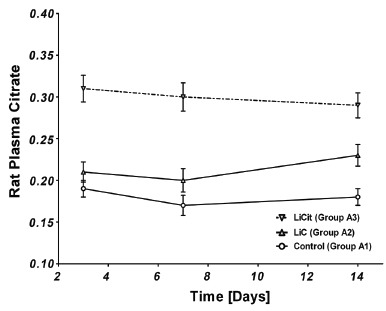
Plasma citrate levels when LiC and LiCit were given in Group A rats. Plasma citrate levels are significantly increased with administration of LiC or LiCit.

**Figure 4:**
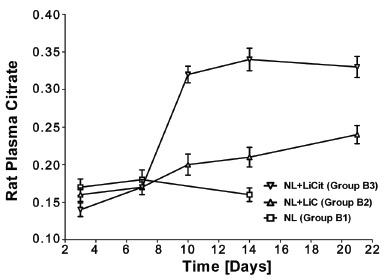
Plasma citrate levels in NL model rats (Group B). Plasma citrate levels increased after a week of Lithium therapy in both Group B2 and B3.

To better characterize the lithium-induced perturbations in citrate handling, plasma concentrations of citrate was measured. The trends in plasma citrate were similar to their urinary levels. In Group A rats, plasma citrate increased immediately for both Li treated sub-groups (Figure 3). Though the levels were lower in animals treated with LiC (Group A2) as compared to LiCit (Group A3), they both demonstrated a statistically significant increase in plasma citrate (*p*<0.001). Similarly, Li treated group B rats (Group B2 and B3) demonstrated a week delay in elevations of plasma citrate (Figure 4), consistent with the delay in its urinary excretion. The increase, though less in NL+LiC as compared to NL+LiCit sub-group, was still statistically significant (*p*<0.001). These results suggest a link between urinary and plasma citrate handling.

### Plasma Lithium Levels

**Figure 5:**
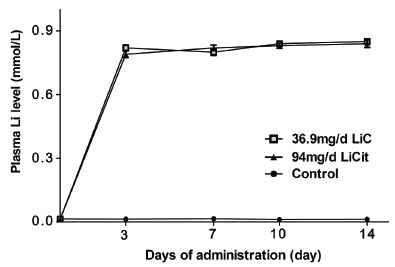
Plasma Lithium levels corresponding to 36.9 mg/d and 94 mg/d of LiC and LiCit respectively as compared to control.

Clinically, lithium is titrated to efficacy and serum measurements are monitored to prevent toxicity. While, the serum levels of 0.8–1.2mmol/L are therapeutic, levels >1.5 mmol/L are considered toxic(16). In these rat studies, the plasma level of lithium never exceeded 1mmol/L (Figure 5). Plasma lithium levels were significantly correlated with the treatment dose of LiC or LiCit, however no obvious further increase was observed after the initial seven days of treatment.

### Effects of LiC or LiCit

**Figure 6:**
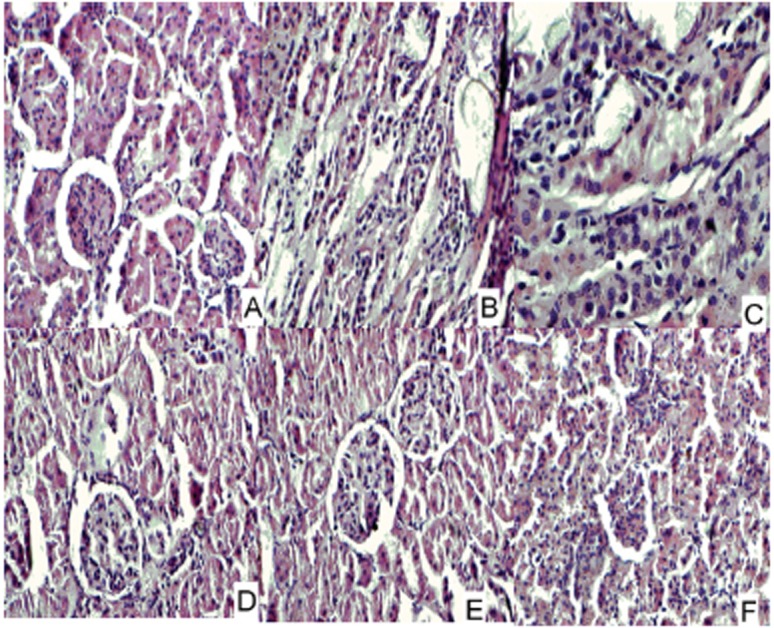
Renal morphological alterations of experimental nephrolithiasis in rats (A), Normal kidney (×200). (B), Renal morphological alterations of rats at 14th day after 5% AmOx contained forge administration, showing obviously dilated renal tubules and marked inflammatory cell infiltration in the tubulointerstitium (×200). (C) and (D), Renal morphological alterations of rats of experimental NL after LiC or LiCi treatment for 7 days, displaying mildly dilated renal tubules and less inflammatory cells infiltration (×200). (E) and (F), Renal morphological alterations of experimental NL rats treated with LiC or LiCi for 14 days respectively, displaying above mentioned lesions caused by AmOx administration were further alleviated.

Urinary oxalic acid (OA) and calcium levels (Ca^++^) were significantly higher in all group B rats irrespective of the lithium treatment while the plasma OA levels (data not shown) were unchanged. In B1 rats, the mean urinary OA was 0.65 ± 0.05 mmol/L (*p*<0.001 vs. control) while mean urinary Ca^++^ was 3.44 ± 0.05 mmol/L (*p*<0.001 vs. control). (Table 2) Crystal deposits that were seen in the renal medulla and papilla in 100% of NL model rats (Group B) on day 7 were left in just 10% of the Lithium treated rats (Group B2 and group B3) on day 14 (*p*<0.05). By day 21, none of the Li treated rats had crystal deposits (Table 3), although oxaluria and crystalluria, crucial factors in NL development, remained higher when compared to control during the course of the treatment (*p*<0.01). Further, grade 2 to grade 3 pathologic alterations that were observed in group B1 (NL) rats were reversed by day 14 in group B2 (NL+LiC) and group B3 (NL+LiCit) (Tables 4, Figure 6).

**Table 2:** Metabolic changes in Serum uric acid (UA) and Urine oxalic acid (OA), pH and calcium (Ca) of animals treated with LiC and LiCit

Item	Con (A1)	LiC (A2)	LiCit (A3)	NL (B1)	NL+LiC (B2)	NL+LiCit (B3)

**UA (mmol/L)**						
3d	3.10 ± 0.85	3.12 ± 0.47	3.11 ± 0.49	3.09 ± 0.59	3.08 ± 0.61	3.06 ± 0.49
7d	3.11 ± 0.66	3.37 ± 0.88	3.23 ± 0.62	3.21 ± 0.69	3.42 ± 0.84	3.27 ± 0.64
14d	3.19 ± 0.75	4.29 ± 1.66	4.59 ± 1.12	3.97 ± 1.14	4.50 ± 1.56	4.79 ± 1.29
**OA (mmol/L)**						
3d	0.19 ± 0.09	0.18 ± 0.05	0.19 ± 0.06	0.59 ± 0.21	0.62 ± 0.26	0.71 ± 0.28
7d	0.21 ± 0.11	0.17 ± 0.04	0.22 ± 0.08	0.69 ± 0.23	0.65 ± 0.19	0.66 ± 0.23
14d	0.20 ± 0.11	0.20 ± 0.10	0.21 ± 0.10	0.66 ± 0.22	0.68 ± 0.24	0.63 ± 0.22
**pH**						
3d	5.60 ± 0.52	7.43 ± 0.40	7.11 ± 0.54	6.06 ± 0.81	6.08 ± 0.55	7.05 ± 0.46
7d	5.52 ± 0.50	7.42 ± 0.55	7.12 ± 0.56	6.17 ± 0.73	6.05 ± 0.45	7.32 ± 0.61
14d	5.50 ± 0.50	7.40 ± 0.35	7.02 ± 0.56	6.17 ± 0.73	7.42 ± 0.44	7.14 ± 0.66
**Ca (mmol/L)**						
3d	2.36 ± 0.50	2.46 ± 0.61	2.51 ± 0.37	3.38 ± 0.38	3.61 ± 0.29	3.46 ± 0.52
7d	2.47 ± 0.46	2.46 ± 0.61	2.44 ± 0.38	3.47 ± 0.41	3.55 ± 0.34	3.39 ± 0.47
14d	2.61 ± 0.32	2.58 ± 0.46	2.43 ± 0.51	3.47 ± 0.41	3.55 ± 0.34	2.79 ± 0.72

There were no significant changes in Serum UA levels among different groups. OA and Ca^++^ were significantly higher in all NL model rats (Group B). pH was higher than controls in Li^+^ treated groups.

**Table 3:** The occurrence ratio of crystalluria and crystal deposits in rat kidney in different groups

Group	N	Crystalluria (%)					Crystal Deposits (%)				
		3d	7d	10d	14d	21d	3d	7d	10d	14d	21d

A1 (Con)	30	0	0		0		0	0		0	
A2 (LiC)	30	0	0		0		0	0		0	
A3 (LiCit)	30	0	0		0		0	0		0	
B1 (NL)	30	80	100		100		20	100		100	
B2 (NL+LiC)	50	80	100	100	100	100	20	100	30	10	0
B3 (NL+LiCit)	50	80	100	100	100	100	20	100	30	10	0

All rats in Group B had crystalluria by day 7. No crystal deposits were observed by day 21 in rats treated with Lithium while they persisted in untreated rats (Group B1). Note: all values are in percentage.

**Table 4:** Pathological changes observed in rat kidney on day 14

Group	N	Dilation of renal tubules				Infiltration of inflammatory cells			
		0+	1+	2+	3+	0+	1+	2+	3+

A1 (Con)	10	10	0	0	0	10	0	0	0
A2 (LiC)	10	10	0	0	0	10	0	0	0
A3 (LiCit)	10	10	0	0	0	10	0	0	0
B1 (NL)	10	0	0	3	7	0	2	4	4
B2 (NL+LiC)	10	7	2	1	0	7	1	2	0
B3 (NL+LiCit)	10	8	2	0	0	9	1	0	0

More injury was observed in Group B rats and this kind of damage was markedly alleviated by LiC or LiCit treatment.

Urinary pH values were increased significantly during and after the treatment with LiC (Group A2) or LiCit (Group A3). Their mean pH value was 7.42 ± 0.02 (*p*<0.001 vs. controls) and 7.08 ± 0.06 (*p*<0.001 vs. controls) respectively (Table 2). Lithium may also affect PCT uric acid handling (17) and hence we investigated its impact on uric acid (UA) excretion. Urinary UA levels in these rats were not significantly increased in any group as compared to the control (Table 2).

## DISCUSSION

The high incidence and prevalence of NL warrants development of new and improved medical therapies. Advances in technology have enabled the treatment of most urinary stone disease without open surgery. Hypocitraturia is the most frequently encountered risk factor, in the development of calcium oxalate stones, accounting for over 60% percent of all NL patients (8). Citrate is a natural inhibitor of NL and multiple studies have demonstrated the utility of citrate supplementation in preventing recurrent stone disease; however, current formulations of citrate supplements are poorly tolerated leading to low compliance (18).

Recently, the physiological regulators of citrate transport have been investigated and characterized (9). These transporters include NaDC-1 and NaDC-3, which regulate citrate reabsorption in the proximal tubules and NaCT which transports citrate into the liver. NaDC-1 exhibits low affinity for its dicarboxylate substrates. The physiological function of NaDC-1 is to absorb Krebs cycle intermediates in the intestine and kidney (19) and is up-regulated in experimental models of NL (20). NaDC-3 is also a Na+-coupled dicarboxylate transporter and exhibits relatively high affinity for its substrates compared to NaDC-1 (13). NaDC-3 is expressed primarily in the basolateral membranes of the epithelial cells in the kidney and intestine (13). In the kidney, NaDC-3 is involved in generating the driving force for the organic anion transporter1 (OAT1) to facilitate the active entry of organic anions into the tubular cells across the basolateral membrane (21). NaCT, a new member of this transporter family is a Na+−coupled citrate transporter (13). It is expressed predominantly in the liver and to a lesser extent in the testes and brain. NaCT is the first plasma membrane transporter described in mammals that functions primarily in the cellular uptake of citrate (13). Interestingly, the sodium binding sites in all these transporters can be competitively bound by lithium. In the case of NaCT this results in enhanced transport activity from the plasma to the liver; however, in the case of the renal tubular transporters, this potentially may result in the inhibition of citrate reabsorption or in effect a urinary “citrate leak” (22). This concept prompted the current study and suggests an entirely new approach of augmenting urinary citrate excretion using lithium.

We investigated the efficacy of LiC and LiCit in regulating rat urinary citrate levels and preventing NL development in an animal model of NL. A variety of methods exists for ensuring calcium based stone formation in the rat. We chose to use administration of high doses of ammonium oxalate (OA, 5%) daily, as it produces a very high oxalate load in the urine and uniformly results in renal tubular deposition easily evaluable under standard polarized microscopy (23, 24). Crystal deposition in renal parenchyma is closely related to kidney stone formation in physiological conditions. Randell's plague described by Randell in 1937 was hypothesized to be a nidus for stone formation. This finding was further concurred by Low and Stroller in 1997 who found that papillary plagues were present in 74% of stone formers as compared to 43% of control subjects.

We observed that NL model rats that were treated with Li salts had a week delay in elevation of urinary citrate levels which correlated with their plasma levels. There could be multiple potential reasons for this effect. For instance, this delay could represent a delay in bioavailability due to the method of induction of the CaOx stones (i.e. from gastro-intestinal binding of the lithium salt to the AmOx solution) or it may be a consequence of the actual nephrolithiasis causing a changes in citrate salts and hence producing deceivingly lower levels of citrate in our essays for initial one week. It should also be noted that the relationship between plasma citrate and urinary citrate levels revealed in this study is counter-intuitive. If the excretion of citrate is increased by lithium, then we would have predicted that plasma levels of citrate would decrease proportionally, not increase as is observed experimentally. The increase is modest, but does reach statistical significance. Such a paradoxical finding suggests increased urinary excretion of citrate is not just a consequence of inactivation of the sodium dicarboxylate co-transporters, but also a consequence of higher plasma levels of citrate. While the citrate present in the lithium citrate salt formulation may have accounted for some of this pronounced effect, its presence in the lithium carbonate arm indicates that this is not just an artifact of increased citrate load. Presumably, the higher citrate levels may also occur because of other lithium sensitive dicarboxylate co-transporters located in other areas. Both urinary and plasma citrate levels in rats treated with LiCit are higher than that with LiC when given an equivalent molar Lithium ion doses, respectively. Since the Lithium ion is thought to be the physiological regulator of citrate transport (22), these differences likely represent the inherent differences in absorption, biodistribution, and bioavailability between the different formulations of the lithium salts.

In our rat model of NL, we observed massive crystal deposition in the renal medulla and the papilla, resulting in dilated renal tubules and marked inflammatory cell infiltration in the tubulo-interstitium after 7 days of administration of AmOx. These lesions were gradually alleviated after LiC or LiCit administration (Table 2 and Table 3) indicating increased citrate level in urine may actually reverse established crystal deposition as well as effectively inhibit the precipitation process. The net effect is a reduction in renal crystal deposition and prevention of downstream effects such as dilation of renal tubules and inflammatory cell infiltration. These findings suggest that there may be benefits to this strategy even in those patients with recalcitrant, recurrent stone disease.

Lithium salts have been widely used clinically for bipolar-affective disorder and have a well established safety profile. The doses of lithium used in our study resulted in serum levels of < 0.9 mmol/L, which is the lower normal therapeutic serum level for bi-polar disorder. At these serum Li levels we observed a 400% to 500% increase in urinary citrate levels after 3 days of administration. There have been several studies determining that the difference in the urinary citrate levels among recurrent stone formers and normal subjects range from approximately 20% to 80% (8, 25–29). This suggests that only a fraction of the dose of Li used for mania would be needed to convert a hypocitraturic recurrent stone former to a normocitraturic individual and thus preventing recurrent nephrolithiasis. We can hence conclude that for treating NL, Li would have a wider clinical utility with little likelihood of side effects.

In conclusion, we describe a new strategy for the medical treatment of NL. Our data indicates that lithium can significantly increase urinary citrate levels, while decreasing NL development in an experimental rat model. It inhibits AmOx induced renal tubular impairment, reduce kidney CaOx crystal accumulation and alleviate the downstream renal tubular injury inherent in stone disease. Given the choice between the two formulations, our studies would suggest that lithium citrate would be the preferred drug as it results in the higher urinary excretion of citrate. These data support the need for clinical trials for the evaluation of the effect of lithium supplementation in preventing recurrent NL.
